# Workplace Determinants of Depression, Anxiety, and Stress in U.S. Mariners during the COVID-19 Pandemic

**DOI:** 10.3390/ijerph192416628

**Published:** 2022-12-10

**Authors:** Ahmad H. Yassin, June T. Spector, Luke Mease, Alice Shumate, Ryan Hill, Jennifer E. Lincoln, Marissa G. Baker

**Affiliations:** 1 Department of Environmental & Occupational Health Sciences, University of Washington, 4225 Roosevelt Way NE, Suite 100, Seattle, WA 98105, USA; 2 Preventive Medicine, Madigan Army Medical Center, Joint Base Lewis-McChord, WA 98431, USA; 3 Western States Division, National Institute for Occupational Safety and Health, Spokane, WA 99207, USA; 4 Office of the Director, National Institute for Occupational Safety and Health, Washington, DC 20201, USA; 5 Division of Safety Research, National Institute for Occupational Safety and Health, Morgantown, WV 26505, USA

**Keywords:** maritime, mariners, stress, depression, anxiety, COVID-19, workplace factors

## Abstract

United States (U.S.) mariners continued sailing throughout COVID-19. Many aspects of their work could make them prone to adverse mental health outcomes but research on workplace determinants of their mental health during COVID-19 is limited. Between January and July 2021 an online survey assessed the outcomes of increased depressive symptoms, increased anxiety symptoms, and increased perceived stress in addition to concerns, worries, and experiences when sailing during COVID-19, job satisfaction, and safety climate in *n* = 1384 U.S. mariners. Demographic measures were also collected. Logistic regression models (for depression and anxiety) and a linear regression model (for stress) were developed. We found that increased COVID-19 concerns and poor self-reported mental health were related to increased odds of likely depression and anxiety and higher stress. Mariners who experienced more adverse experiences aboard a vessel had increased stress and increased odds of depression. Poor sleep quality was also related to increased odds of depression, and poor vessel support/safety culture was related to higher stress. Differences in outcomes were seen by vessel type, age, and credential in regression analyses. Results from this study will help to prioritize interventions to minimize the mental health impacts of COVID-19, and influence evidence-based recommendations to improve the mental health of mariners going forward.

## 1. Introduction

The COVID-19 pandemic profoundly impacted work environments and workers. In addition to increasing risk of infection for those workers who could not work from home [1], the literature reports that COVID-19 also impacted the mental health and stress of workers, due to concerns about infection of self and family, worry about job and financial security, increased job demands, interactions with the public, and changes in workplace policies and protocols due to COVID-19, amongst other workplace factors [2,3,4,5]. Across occupations and industries, mental health and stress have always been closely linked with work, with aspects such as scheduling/hours, workload/pace, working conditions, support from co-workers and employers, experiences of violence, harassment or discrimination, conflicting home/work demands and other factors related to worker mental health and stress [6,7]. Many of these factors could be exacerbated due to the COVID-19 pandemic.

COVID-19 also brought an increased awareness of occupations that are essential to continuing vital infrastructure operations. United States (U.S.) mariners are essential workers who play a vital role in transporting goods and people throughout the U.S. and worldwide. During the COVID-19 pandemic, maritime industry and public health officials enacted changes to policies and protocols to halt the transmission of SARS-CoV-2 for mariners on vessels [8,9]. In the “State of the U.S. Maritime Industry: Impacts of the COVID-19 Pandemic” Congressional Hearing, mariners and maritime industry representatives reported facing lockdowns, mandatory quarantines, and travel and port restrictions imposed by governments around the world [10]. These circumstances often restricted crew changes and made the repatriation of seafarers challenging, raising concerns regarding the mental and physical safety of mariners stuck onboard vessels for longer than their planned afloat tours. The abandonment of seafarers internationally also arose during the pandemic, and many mariners experienced long periods of time on vessels without access to shore leave or the ability to return home, which could increase the social isolation many mariners already experience [10,11]. Even during non-pandemic times, mariners face a host of occupational safety and health hazards on the vessel, adverse weather, lack of access to family and loved ones, isolation on board, musculoskeletal disorders, and conflicts resulting from living and working in close quarters with other mariners [12,13,14,15,16]. However, there are various maritime jobs; while some mariners may spend weeks or months at sea on oceangoing vessels, others may work on day boats, such as operating a ferry or harbor tug, and others may work on a government research or response vessel, all which could result in differential occupational health hazards.

Many of the workplace conditions routinely experienced by mariners could increase their susceptibility to adverse mental health outcomes and high stress, with the changes and stresses due to the COVID-19 pandemic further increasing likelihood. There has been limited academic research into the physical and mental health of U.S. mariners, with this small body of literature limited to research conducted before COVID-19 pandemic [17]. Looking specifically at U.S. mariners in their sample of pilots and masters, Lefkowitz et al., investigated potential workplace factors and found that company culture, social isolation, violence at work, job dissatisfaction, and extended tours were associated with increased likelihood of depression and anxiety [17]. Scovill et al., investigated physical health characteristics of U.S. mariners (inland waterway captains and pilots), finding a high prevalence of obesity, smoking, high blood pressure, and other markers of metabolic syndrome [18].

More academic literature has looked at mental health and stress in seafarers that do not sail under U.S. flagged vessels (international seafarers), both before the COVID-19 pandemic [19,20,21,22,23,24,25] and during the COVID-19 pandemic [26,27,28,29]. In a matched sample analysis of international seafarers, Pauksztat et al., found significantly higher depression and anxiety scores in mariners surveyed during the pandemic, compared to before the pandemic, and higher scores were associated with longer work periods, a longer time than expected spent aboard a vessel, and being on a vessel with a “flag of convenience”, the latter of which would not apply to U.S. mariners [26]. Similarly, in a survey administered during COVID-19 to *n* = 439 multinational seafarers, who often spend long periods of time on vessels than U.S Mariners, Baygi et al., found that the duration of stay on a vessel was related to increased odds of depression, and non-officers experienced fewer symptoms of anxiety and depression than officers [27,28]. In work specific to Chinese seafarers during the early months of COVID-19 (June–July 2020), Qin et al., found increased odds of depression to be associated with less leisure time or physical exercise, poor sleep quality, longer work hours, and higher perceived work stress [29].

Being mentally healthy is important for mariners given the mentally and physically demanding nature of their job, and the need to work as a cohesive crew. As poor mental health has been found to be related to injury at work [30,31], burnout [21,32], poor decision-making [33], and attrition [3,34,35] across a range of occupations, there is industry motivation to describe the burden of mental health outcomes of U.S. mariners and to develop interventions to improve mariner mental health, especially given the added changes and stressors faced by the industry due to COVID-19. Therefore, characterizing the mental health needs of mariners is of top priority for public health, and the maritime industry.

Through the collaboration of federal and state partners and industry leaders, we investigated workplace factors related to depression, anxiety, and stress in a survey of *n* = 1384 U.S. Coast Guard credentialed mariners who actively sailed during the COVID-19 pandemic. Underpinning this survey were the hypotheses that workplace determinants and factors caused or exacerbated by the COVID-19 pandemic were impacting the mental health and stress of U.S. mariners, and that certain groups of mariners (defined by demographic or occupational factors) may be more likely to experience symptoms related to depression, anxiety, or stress. To investigate these hypotheses, we had two primary aims for our survey:

(1) Determine the number and prevalence of mariners in our sample reporting symptoms of depression, anxiety, and stress, and investigate whether the prevalence differs by demographic and occupational factors;
(2) Characterize workplace and personal factors arising from COVID-19 that could impact mariners, and whether these factors relate to the likelihood of depression, anxiety, or symptoms of stress in our sample.

Results from these aims can be used to identify areas of intervention for the industry to improve mariner mental health during public health emergencies and beyond. The work presented here adds to the growing body of literature characterizing the impact of COVID-19 on essential workers and how workplace determinants influence worker mental health. Our study is the first to investigate how workplace factors and experiences specific to COVID-19 impacted self-reported symptoms of depression, anxiety, and perceived stress of U.S. mariners. The work summarized here is important for identifying ways to support mariners’ wellbeing and will increase our body of knowledge related to how the workplace can influence mental health and wellbeing.

## 2. Materials and Methods

The study population of interest was U.S. Coast Guard credentialed mariners who sailed during the COVID-19 pandemic (since 1 March 2020). This study was a collaboration between University of Washington and researchers from the National Institute for Occupational Safety and Health (NIOSH), guided by a working group consisting of representatives from the U.S. Committee on the Marine Transportation System (CMTS) COVID-19 Working Group, the U.S. Department of Transportation Maritime Administration (MARAD), and the Ship Operations Cooperative Program (SOCP). The University of Washington Human Subjects Division determined this project to be exempt from review, as researchers were collecting no identifying information.

### 2.1. Survey Development

A web-based survey was developed in REDCap [36] to collect information on workplace experiences and concerns specific to mariners, mental health outcomes, and workplace factors such as safety climate and support. A working group consisting of University of Washington and NIOSH researchers as well as representatives from the external advisory group co-developed the survey, which was informed by mental health surveys deployed by investigators in other occupational cohorts during COVID-19 [30,32], as well as industry needs and previously validated scales.

The survey went through two separate rounds of pilot testing with 30 individuals, comprising members of the study population and those with maritime industry expertise, to ensure the survey used appropriate language, was clear, all skip logic worked, and it took less than 15 min to complete, on average. After integrating feedback from pilot testers, the online survey was open to all U.S. mariners for six months, from 25 January 2021 through 31 July 2021. The survey was promoted to mariners by employers, unions, U.S. Coast Guard, and other professional organizations using email, websites, trade publications, and flyers, directing mariners to the website to take the survey. The survey received a total of *n* = 1686 responses, and *n* = 1384 of respondents met study criteria (having actively sailed during the COVID-19 pandemic and were U.S. Coast Guard credentialed) and were retained for analysis. All questions on the survey, except those to assess inclusion criteria, were optional.

### 2.2. Survey Metrics Collected

Broadly, the survey assessed workplace experiences and worries during COVID-19, safety climate and job satisfaction, outcomes related to mental health and stress, and demographic factors. The survey used validated scales to assess two mental health outcomes, as well as stress.

#### 2.2.1. Outcome Measures

Depression was assessed using the two-question Patient Health Questionnaire (PHQ-2) [37], anxiety was assessed using the two-question General Anxiety Disorder 2-Item (GAD-2) [38], and perceived stress was assessed using the four-question Perceived Stress Scale (PSS-4) [39]. For these outcome measures, individual questions in the scale are added together (two questions in the PSS-4 are reverse coded) and a higher score indicates increased stress (PSS-4), or increased likelihood of generalized anxiety disorder (GAD-2) or major depressive disorder (PHQ-2). PHQ-2 and GAD-2 scores range from 0 to 6 and PSS-4 scores range from 0 to 16. Both PHQ-2 and GAD-2 have validated clinical cut-off scores, with a total score ≥ 3 indicating clinical depression or anxiety is likely and further clinical evaluation is warranted [37,38]. PSS-4 does not have a validated cut-off score, but a score ≥ 6 has been used to indicate higher perceived stress in other cohorts [40].

#### 2.2.2. Workplace Determinants of Mental Health and Stress during COVID-19

##### Job Satisfaction

The survey used three questions to evaluate job satisfaction, adapted from Spector’s 36-question Job Satisfaction Scale [41]. Job satisfaction questions asked respondents how much they agree (1 = strongly disagree, 2 = disagree, 3 = agree, 4 = strongly agree) with the statements: “I like the people I work with”; “I like doing the things I do at work”; and “I get enough time to relax and recharge when on the vessel.” Raw scores for all three questions were averaged for a composite job satisfaction score; higher scores indicated more job satisfaction. When combining the three questions, the overall job satisfaction had a Cronbach’s alpha (Cα) of 0.67 (CI: 0.64, 0.70), indicating acceptable reliability between the measures.

##### Mariner Concerns during COVID-19

Questions specific to mariner concerns, experiences, and scheduling/contract impacts during COVID-19 were developed. A composite scale to summarize mariner concerns during COVID-19 was based on seven questions, where mariners were asked to assess how often (1 = never, 2 = almost never, 3 = sometimes, 4 = fairly often, 5 = very often) they had been concerned about the following when on a vessel during COVID-19: being away from family; mariner contract length; money; lack of work; a family member getting COVID-19 while I am away; myself getting COVID-19 while at sea or ashore; extra work onboard if there is a COVID-19 case on my vessel. For respondents who answered all seven questions, raw scores were averaged with a higher score indicating more concerns when sailing during COVID-19. Combining the seven questions resulted in a Cronbach’s alpha (Cα) = 0.74 with a 95% CI: 0.71, 0.77.

##### Mariner Experiences during COVID-19

A composite scale to summarize mariner experiences during COVID-19 was based on seven questions, where mariners were asked to assess how often (1 = never, 2 = almost never, 3 = sometimes, 4 = fairly often, 5 = very often) they had experienced the following: having no access or inadequate access to Internet on a vessel; having no cell phone or inadequate cell phone service while in port, harbors, and inland waterways; being restricted in my ability to join or rejoin my family; having feelings of isolation aboard; having to quarantine due to potential exposure to COVID-19; being denied shore leave when in port due to the COVID-19 pandemic; being unable to join a vessel due to interruption of normal crew changes during COVID-19. Raw scores were averaged, and a higher score indicated more adverse experiences when sailing. Combining these seven questions resulted in a Cronbach’s alpha (Cα) = 0.86 with a 95% CI: 0.85, 0.87.

##### Impacts on Mariner Vessels or Contracts during COVID-19

Mariners were asked how their vessel or mariner contract had been impacted by the COVID-19 pandemic, by responding (yes/no) if the following had happened: personnel has been infected with COVID-19 on my vessel; port calls have been affected; cargo onload/offload has been affected; shore leave has been canceled; shore medical visits have been impacted; my mariner contract has been extended voluntarily; my mariner contract has been extended involuntarily; I have had restricted ability to get on or off the vessel. The total number of impacts was summed for each respondent and is reported as a 0 to 8 score, with a higher score indicating that mariners had more impacts on their contract or vessel during the pandemic. Mariners were also asked how long they had been on a vessel without access to shore leave, since March 1, 2020 (1 = Less than 2 months, 2 = 2 to 4 months, 3 = 4 to 6 months, 4 = More than 6 months).

##### Safety Climate and Support

Safety climate and support was assessed separately for on the vessel and from organization management. For the vessel safety climate and support scale, respondents were asked to rate their level of agreement (1 = strongly disagree, 2 = disagree, 3 = agree, 4 = strongly agree) with three statements: if I am feeling sad or stressed there is usually someone on the vessel I can talk to; senior vessel officers encourage the crew to get mental health help if needed; and senior vessel officers generally encourage employees to work in accordance with safety rules, including COVID-19 protocols. For the management safety climate and support scale, respondents used the same agreement scale to rate their agreement with two questions: management at my organization generally encourages employees to work in accordance with safety rules, including COVID-19 protocols, and management at my organization encourages the crew to get mental health help if needed. For both scales, questions were averaged for a composite score, with the vessel support scale having a Cronbach’s alpha (Cα) = 0.67 with a 95% CI: 0.64, 0.70 and the management support scale having a Cronbach’s alpha (Cα) = 0.74 with a 95% CI: 0.71, 0.77.

#### 2.2.3. Personal and Demographic Characteristics of Respondents

Respondents were asked to self-rate their mental health, physical health, and sleep quality using a five-point scale (1 = poor, 2 = fair, 3 = good, 4 = very good, 5 = excellent), and reported the number of average days each week they get at least 30 min of physical activity, outside of work activities. Demographic and background variables collected included age, gender, race/ethnicity, credential, and the maritime industry in which the respondent works. Any group that had fewer than 10 responses was not presented in summary tables, and was either grouped into another category (e.g., race/ethnicity groups with small numbers were grouped into “other race/ethnicity or multiple race/ethnicity”) or dropped from presentation on stratified tables (e.g., genders other than male or female were not presented in stratified analyses due to small sample size, but were included in regression models). “Prefer not to answer” was a survey option for age, gender, and race/ethnicity; when a respondent skipped the question without checking any box it was coded as “Missing”.

### 2.3. Survey Data Analysis

Raw data were downloaded from REDCap. Questions were scored and combined to create scales, as explained above. When questions were combined into a scale, some were reverse coded to ensure all questions were either in the affirmative or negative to ease interpretation of the scale and results. Descriptive statistics and analysis of variance (ANOVA) were used to characterize and compare PHQ-2, GAD-2, and PSS-4 scores between groups defined by demographics, industry, and credentials. A Venn diagram was produced to quantify and visualize the number of respondents who had a PHQ-2 score ≥ 3, GAD-2 score ≥ 3, and PSS-4 score ≥ 6 (the population average), and multiple or all.

Logistic regression models were developed for the outcomes of PHQ-2 and GAD-2; a linear regression model was developed for PSS-4. All models included the composite scales of vessel COVID-19 concerns, vessel experiences, mariner contract impacts, vessel safety support, management safety support, and job satisfaction. Time without shore leave, self-reported physical health, self-reported mental health, self-reported sleep quality, and weekly physical activity were also included as predictors. All models included age, credentials, and vessel. Gender and race/ethnicity were not included in models due to sample homogeneity. Statistical significance was defined as *p* ≤ 0.05. All data analyses were completed in R Studio version 2022.02.0.

## 3. Results

A total of 1384 respondents were retained for analysis, but not all questions were answered by all respondents. Table 1 summarizes the demographics and background of these respondents. Of respondents who clicked any demographic answer, respondents predominantly identified as male (85.8%), white (75.6%), and between the ages of 22–64 (85.6%). The majority of the respondents were licensed deck/engineer officers (67.6%). Inland and coastal cargo vessels/workboats (36.6%), government/public vessels (27.1%), and oceangoing cargo vessels (22.0%) were the most common vessels of the respondents.

To inform our first aim, we tabulated mean PHQ-2, GAD-2, and PSS-4 scores, and the percent of respondents with a high score for all respondents and investigated differences by age, gender, race/ethnicity, maritime industry, and credential as presented in Table 2. For all respondents, 20.4% had a PHQ-2 score considered high (major depressive disorder is likely), 22.4% had a GAD-2 score considered high (generalized anxiety disorder is likely), and 37.9% of respondents had a PSS-4 score above the population norm (≥6). As we hypothesized, we saw differences in the outcomes by demographic and occupational characteristics. For all three outcomes, there was an apparent association with age, with older mariners having lower PHQ-2, GAD-2, and PSS-4 scores, and analysis of variance (ANOVA) found statistically significant differences in all three outcomes between groups defined by age. Females tended to have higher PHQ-2, GAD-2, and PSS-4 scores, and more females had a GAD-2 and PSS-4 score considered high. While these differences were not statistically significant between three groups using an ANOVA, when using a *t*-test to compare mean outcome scores just between males and females in the sample, there was a significant difference between males and females for mean GAD-2 score (*p* = 0.0003) and mean PSS-4 score (*p* = 0.02) but not PHQ-2 score (*p* = 0.30). PHQ-2 and PSS-4 scores differed significantly between vessel types, with mariners working on government/public vessels having higher scores for all three measures. No significant differences in outcomes were seen in groups defined by credential. For groups defined by race/ethnicity, significant differences in PSS-4 score were present both when including and excluding the “prefer not to answer group”; no significant differences were seen for PHQ-2 or GAD-2 by race/ethnicity.

**Figure 1:**
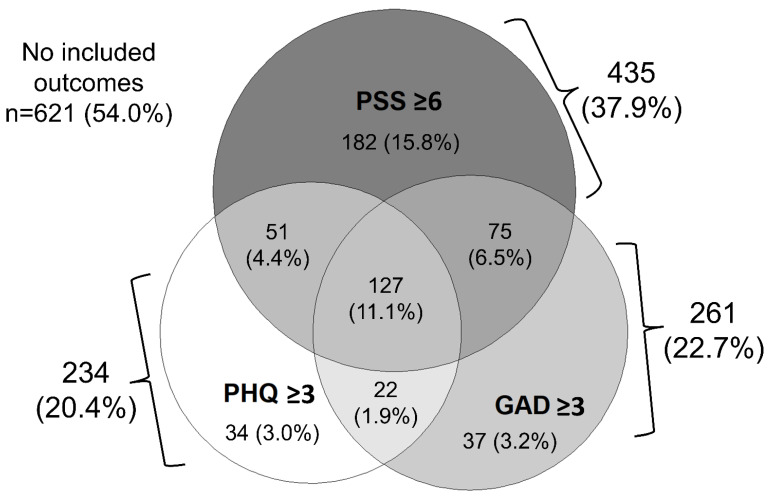
This Venn diagram shows the number of respondents who had one or more of the outcomes considered in this study. Of the *n* = 1149 respondents who had complete scores for all three outcomes, 528 respondents (46%) had at least one of the following: a PHQ-2 score ≥ 3, GAD-2 score ≥ 3, or PSS-4 score ≥ 6, and 11.1% of respondents had all three.

Further informing our first aim, Figure 1 is a Venn diagram showing the overlap between these three outcomes in the population of *n* = 1149 mariners for whom all three outcomes were complete. From this Venn diagram a high PSS-4 score was common in respondents who also had a high PHQ-2 or GAD-2 score. While 54.0% of mariners for whom all outcomes were complete had no high scores, 46.0% had a high score for at least one of the outcomes, and 11.1% had high scores for all three outcomes.

Descriptive statistics for the composite scales and all questions included in the composite scales, as well as other variables included in the regression analyses, are presented in Table 3. These are the workplace determinants that inform our second aim. On average, the top concerns of mariners were being away from family and a family member getting COVID while they were away. The most common adverse experience reported by mariners was being denied shore leave when in port, and while most mariners in our sample (47.1%) went less than two months without access to shore leave, nearly a quarter (23.9%) were on a vessel for more than 4 months without access to shore leave. For mariner impacts, 61.9% of respondents indicated they had restricted ability to get on or off the vessel, and 55.4% of respondents indicated shore leave had been cancelled due to the COVID-19 pandemic. Mariners tended to agree that senior officers and management encourage employees to work in accordance with safety regulations and COVID protocols, and mariners in our sample tend to like the people they work with and the things they do at work. While mariners report good physical and mental health, they report slightly lower sleep quality, on average.

Table 4 summarizes the results of the logistic regressions (for PHQ-2 and GAD-2) and linear regression (for PSS-4), which support our second aim. Results from the logistic regression indicate that respondents working on a government or public vessel were significantly more likely to have a PHQ-2 score ≥ 3 relative to those working on oceangoing cargo vessels (OR: 1.74, 95% CI: 1.04, 2.95). As the frequency of concerns around COVID-19 increased, and the frequency of adverse vessel experiences increased, respondents were significantly more likely to have a PHQ-2 score ≥ 3 (OR: 1.44, 95% CI: 1.06, 1.95 and OR: 1.62, 95% CI: 1.22, 2.17, respectively). Both as self-reported mental health and sleep quality improved, the odds of a PHQ-2 score ≥ 3 significantly decreased (OR: 0.38, 95% CI: 0.29, 0.49 and OR: 0.63, 95% CI: 0.49, 0.81, respectively).

For GAD-2, as age increased, the odds of a GAD-2 score ≥ 3 significantly decreased (OR: 0.87, 95% CI: 0.76, 0.99). As the frequency of COVID-19 concerns increased, the odds of anxiety significantly increased (OR: 1.93, 95% CI: 1.43, 2.61). As self-reported mental health improved, the odds of a GAD-2 score ≥ 3 significantly decreased (OR: 0.27, 95% CI: 0.21, 0.36). While not significant, as time with shore leave increased, the odds of a GAD-2 score ≥ 3 tended to increase (OR: 1.27, 95% CI: 0.99, 1.63).

In the linear regression model for PSS-4, the average PSS-4 score was higher in all vessel types relative to oceangoing cargo vessels. As the frequency of COVID-19 concerns and vessel experiences increased, there was a significant increase in the average PSS-4 score (coef: 0.54, 95% CI: 0.30, 0.77, and coef: 0.30, 95% CI: 0.06, 0.53). As vessel support and safety culture increased, a significant decrease in average PSS-4 was apparent (coef: −0.45, 95% CI: −0.79, −0.10). Like the other outcomes, self-reported mental health was also significantly associated with a decrease in PSS-4 score, with a one-point improvement in self-reported mental health associated with an average decrease in PSS-4 score of −1.25 (95% CI: −1.44, −1.05). These findings confirm our hypothesis that symptoms of mental health and stress were related to workplace determinants and workplace factors caused by COVID-19 in this study population.

Table 5 presents variables included in the COVID-19 concerns composite score and how each variable relates to PHQ-2, GAD-2, and PSS-4 in models including age, vessel type, and credential. Results presented here show that concerns around being away from family, mariner contract length, money, and lack of work are significantly related to mental health outcomes or stress in this population.

## 4. Discussion

U.S. mariners are critical infrastructure workers who continued to work during the COVID-19 pandemic, often with substantial changes in their workplace policies and protocols, put in place to protect mariners and members of the public they may interact with. Our survey sought to characterize how the COVID-19 pandemic had impacted the work of mariners, and how workplace factors may influence depression, anxiety, and stress in U.S. mariners. In our sample, 20.4% of mariners had a PHQ-2 score indicating depressive disorder is likely. In the survey of *n* = 233 U.S.-based masters and pilots undertaken before the COVID-19 pandemic, Lefkowitz et al., found 16% of respondents to have mild depressive symptoms, assessed from the longer PHQ-9 [17]. While there are differences in our population and in how depression was assessed, it is worth noting that depression was of concern prior to the COVID-19 pandemic in this population, and its prevalence may have increased with the additional stressors resulting from the pandemic.

Pauksztat et al., investigated how the COVID-19 pandemic impacted the mental health of international seafarers, using matching samples from before and during the pandemic [26]. Utilizing GAD-2 and PHQ-2, Pauksztat et al., found that the mean scores on both screening tools increased significantly, with a mean PHQ-2 score during the pandemic of 2.50 (SD: 1.03). As shown in Table 2, the mean PHQ-2 score in the U.S. mariner cohort was 1.46 (SD: 1.65), and the mean GAD-2 score was 1.52 (SD: 1.73) which was also lower than the mean GAD-2 score Pauksztat et al., found (mean: 2.63, SD: 1.17). Pauksztat also investigated potential interventions related to mental wellbeing during COVID-19 and found mental health was improved with access to more support on and off the vessel and a strong Internet connection [42]. Similarly, we found that stress was lower in U.S. mariners with higher levels of vessel support, and both the PSS-4 score and the odds of depression were higher in respondents who experienced more adverse vessel experiences, including poor Internet connection.

Using different screening tools to assess depression, anxiety, and stress, Baygi et al., reported a 12.3% prevalence of depression, 11.6% prevalence of anxiety, and 5.9% prevalence of stress in 470 international seafarers working on oil tankers during COVID-19 [28]. Qin et al., found 41.7% of Chinese mariners in their cross-sectional study to have at least mild depressive symptoms, and similar to findings presented in Table 3, found sleep quality to be associated with depression [29]. Taken together, while there is variability in how mental health outcomes are measured and which seafarer populations are surveyed, there is converging evidence of the mental health impact the COVID-19 pandemic has had on seafarers, whether they are working internationally or are U.S. credentialed mariners. However, it must be acknowledged that mariners on international fleets and U.S. fleets may face different stressors due to differences in tour of duty length, distance from home, communication and support available on board, and differences in training programs before sailing. There is also evidence that while the prevalence and likelihood of mental health outcomes may have increased in mariner populations during the pandemic, mental health concerns have likely always been present in the industry and should remain a priority moving forward.

Findings in our study indicate there are several workplace factors related to depression, anxiety, and stress in our U.S. mariner respondents. Increased frequency of COVID-19 concerns (specifically worrying about being away from family, mariner contract length, money, and lack of work significantly increased odds of anxiety and depression in our study, and significantly increased PSS-4 score when controlling for age, industry, and vessel type. This indicates that these stresses unique to COVID-19 influenced the mental wellbeing of U.S. mariners.

Self-reported mental health was associated with depression, anxiety, and stress as assessed using a validated screening tool, which indicates that mariners may be aware of how they are doing mentally and could therefore know when they would benefit from taking advantage of mental healthcare if it is accessible and promoted. Poor sleep quality was also found to increase the odds of depression, though the directionality of all relationships presented here cannot be ascertained from this cross-sectional study.

Differences were also seen between credential and type of vessel, with mariners sailing on government or public vessels being at increased odds of depression and having significantly higher stress scores relative to other vessel types. In bivariate analyses (Table 2) mariners working on passenger vessels had lower PHQ-2, GAD-2, and PSS-4 scores than other vessel types, but also had a very small sample size relative to the other vessel types included in this analysis. However, given that mariners on passenger vessels tend to have a “day job” and do not live on their vessels, work disruptions due to COVID-19 may have been different from those working on oceangoing or inland cargo vessels.

While gender was not included in regression analyses due to the homogeneity of the sample, findings, as presented in Table 2, indicate that mariners identifying as female may have higher rates of anxiety and stress than male mariners, though the number of female-identifying mariners who responded to this survey is small (*n* = 105), so results must be interpreted with caution. Given the minority status women mariners face in the maritime industry and the gendered stressors and harassment faced by women in male-dominated industries [43], this could contribute to the increased rate of mental health outcomes experienced by female mariners in this small sample. In a sample of 595 international female seafarers, Stannard et al., found that 43% of respondents selected stress/depression/anxiety when asked to select the top three health challenges they have; 17% of respondents indicated sexual harassment is an issue for them onboard, though this percentage was found to be higher for women in non-supervisory roles [43]. Together, these findings indicate a need for future research specific to the gendered stressors experienced by female mariners.

In bivariate analyses, younger mariners in this sample (particularly ages 25–34) tended to have higher PHQ-2, GAD-2, and PSS-4 scores, with scores consistently decreasing as age increased. This could be due to increased stressors related to family rearing faced by younger mariners, or be due to a healthy worker bias, since mentally fit mariners would be more likely to stay in the industry into their 50s and 60s.

### 4.1. Recommendations

Results from this study indicate several areas where the maritime industry can intervene to protect and improve the mental health of mariners, even after the COVID-19 pandemic. Industry recommendations include ensuring that mariners have access to mental healthcare even while they are aboard a vessel and that steps are taken to ensure there are no barriers to accessing care on a vessel. This could include having access to phone or Internet-based mental healthcare that can be accessed at all times of day, or even developing app-based modules or training focused on mental health that could be accessed without Internet or a phone. Ensuring mariners have both time to seek mental healthcare and a private space to do so would also be beneficial in encouraging mental healthcare uptake. Seafarer welfare services offer support to both domestic and international mariners across North America, and their services could be valuable to mariners in need of counseling services, or other supports.

Findings from this survey indicate increased training around mental health could also be beneficial to U.S. mariners. Regular and appropriate training could serve to help decrease any stigmas that may be held related to mental health and give mariners tools to not only identify mental health symptoms in themselves but also in others on their vessel.

In particular, efforts should be made to ensure females, racialized, and younger mariners have access to mental health care, in order to ensure that these groups feel welcome in the maritime industry. This will not only improve the occupational health of the workforce, but also improve retention and recruitment efforts.

Mental health surveillance efforts in the U.S. maritime industry should continue, to track the mental health of the industry over time, to validate whether industry interventions have an impact on mariner mental health, and to continue to target interventions to the most at-risk groups. Ensuring that COVID-19 concerns such as those related to money, scheduling, and lack of work do not persist could be beneficial for mariners’ mental health, as well as promoting appropriate sleep hygiene, even if that means changing the schedules that mariners work. These interventions could have the short-term impact of improved worker wellbeing, as well as the long-term impact of reduced injury and illness, and increased retention in the industry. Maintaining mariner mental health is crucial not only for the occupational health of this critical industry, but also for maintaining global and domestic supply chains.

### 4.2. Limitations

There are several limitations to the work presented here which must be acknowledged. As this was a cross-sectional survey, the directionality between mental health outcomes and predictors cannot be determined. This survey is also specific to the mariners who took the survey, which could have been influenced by sampling bias given that mariners with an interest in mental health may have been more likely to participate. Similarly, as we relied on industry partners to promote our survey, it is unknown if efforts to recruit survey respondents were consistent across the industry. This survey was collected from January 2021 to July 2021, a time period when vaccines for COVID-19 were being rolled out to the general public (maritime workers were not prioritized for vaccines in most states’ vaccine distribution plans). Therefore, the changing nature of the pandemic over the six-month period these survey data were collected must be acknowledged, as that could influence mental health/stress, and work practices/policies. This survey was not able to reach many female mariners and racialized mariners to allow us to look at how working during the pandemic impacted these minority populations, though the subcategories are representative of the demographic breakdown in the mariner population as a whole. Future work must prioritize characterizing the experiences of all genders, races, and ethnicities in the workplace with sufficient sample size to best identify most at-risk groups, prioritize interventions, and make conclusions about these groups. Additionally, this survey only characterized stressors related to the workplace, and questions were not asked about stressful events mariners may have experienced in their personal lives that could impact their overall mental health and wellbeing. Questions also were not asked about physical health outcomes, injuries, and behavioral health which could have given a richer context to the experiences of mariners during COVID-19 and areas for intervention. This survey lacks generalizability outside of the sampled population during the time period of the survey, and missing data must also be acknowledged, which may be due to the sensitive nature of the survey questions or respondents stopping the survey midway through due to length or disinterest in the topic.

## 5. Conclusions

To our knowledge, this is the only survey to look at the mental health and stress of U.S. mariners during the COVID-19 pandemic. We found that increased COVID-19 concerns and poor self-reported mental health were related to increased odds of depression and anxiety, and higher stress. More adverse vessel experiences were related to increased stress and increased odds of depression. Poor sleep quality was also related to increased odds of depression, and poor vessel support and safety culture were related to a higher stress score. In regression analyses, increased age was significantly associated with a decreased likelihood of anxiety, and mariners working on government/public vessels had a significantly higher likelihood of depression and significantly higher PSS-4 score relative to mariners on oceangoing cargo vessels. Licensed deck or engineer officers had a significantly decreased likelihood of anxiety relative to cadets/pilots. In bivariate analyses, significant differences in PSS-4 score were seen by race/ethnicity, though sample sizes for categories other than white were very small and therefore findings must be interpreted with caution.

Results from this study inform both practical and theoretical implications. Results presented here will have direct value to those in the maritime industry and could inform evidence-based policies or practices around mental healthcare delivery, access, or awareness which will improve the mental health of mariners going forward, as well as make the industry more attractive to incoming mariners. Of the *n* = 1149 respondents who had complete scores for all three outcomes, 46% of respondents had a high score for at least one of the outcomes, and 24% of respondents had a high score for two or all outcomes. This indicates that the most effective workplace mental health interventions will be comprehensive to address multiple outcomes, and any workplace mental health assessment or evaluation should investigate multiple mental health outcomes, since it is not uncommon for workers to screen positive for multiple.

Additionally, this study further informs the relationship between work and mental health, and how profoundly COVID-19 impacted the U.S. maritime industry. This work also underscores the need for additional research to focus on the specific needs of racialized and female mariners, with an appropriate sample size to inform actionable conclusions and an exploration of stressors that may be unique to minority mariner populations. The maritime industry should take steps to improve mental health and decrease stress in their mariners, including ensuring there is access to mental healthcare for mariners both at home and on vessels, and that mental health outcomes are systematically tracked by the industry with findings acted upon. Ensuring mariners receive appropriate and frequent communication and training on mental health topics is also warranted. Improving sleep hygiene through both individual and industry actions and focusing on exploring the mental health needs of younger (particularly 25–34 years of age) and minority mariners (including women, and racialized mariners) could have positive impacts not only for mariner wellbeing but also industry recruitment and retention [40,43]. Taking steps to decrease mariner concerns around things such as money, lack of work, and contract length are further changes that the industry could adopt to improve mariner mental health. While these data were collected between January–July 2021 during the COVID-19 pandemic, findings here are still important for understanding how experiences, concerns, and stressors unique to a U.S. maritime population impact their mental health and wellbeing, and to help to identify areas of intervention for this critical workforce.

## Figures and Tables

**Table 1:** Characteristics of respondents.

		*n*	% (Total) *	% (Responses) **
**Age (years)**	18–24	52	3.7%	4.8%
	25–34	241	17.4%	22.4%
	35–44	232	16.7%	21.6%
	45–54	194	14.0%	18.0%
	55–64	254	18.3%	23.6%
	65+	68	4.9%	6.3%
	Prefer not to answer	35	2.5%	3.3%
	Missing	313	22.5%	--
**Gender**	Male	918	66.1%	85.8%
	Female	105	7.6%	9.8%
	Prefer not to answer	47	3.4%	4.4%
	Missing	314	22.6%	--
**Maritime Vessel**	Oceangoing cargo vessels	305	22.0%	22.0%
	Inland and coastal cargo vessels/workboats	507	36.5%	36.6%
	Passenger vessels	88	6.3%	6.4%
	Government/public vessels	375	27.0%	27.1%
	Other vessels	109	7.8%	7.9%
	Missing	5	0.4%	--
**Position**	Cadet/Pilot	75	5.4%	5.5%
	Licensed deck/engineer officer	928	66.8%	67.6%
	Unlicensed deck/engineer	228	16.4%	16.6%
	Other position	142	10.2%	10.3%
	Missing	16	1.2%	--
**Race/Ethnicity**	American Indian or Alaska Native	18	1.3%	1.7%
	Asian	21	1.5%	1.9%
	Black or African	24	1.7%	2.2%
	Hispanic, Latinx, Spanish origin	25	1.8%	2.3%
	White	815	58.7%	75.6%
	Other race/ethnicity or multiple race/ethnicity	82	5.9%	7.6%
	Prefer not to answer	93	6.7%	8.6%
	Missing	311	22.4%	--

* % Of respondents including data characterized as missing. ** % Of respondents when excluding data characterized as missing

**Table 2:** Distribution of outcomes by demographic factors.

		PHQ-2 Score					GAD-2 Score					PSS-4 Score			
		Range: 0–6					Range 0–6					Range 0–16			
Group	*n*	Mean PHQ-2 Score	(SD)	% High	p *	*n*	Mean GAD-2 Score	(SD)	% High	p *	*n*	Mean PSS-4 Score	(SD)	% High	p *
**Age **															
18–24	50	1.46	(1.42)	22.0%	<0.001	51	1.59	(1.79)	29.4%	<0.001	50	5.00	(3.06)	40.0%	<0.001
25–34	241	1.90	(1.76)	27.8%		241	2.09	(1.88)	36.9%		239	5.52	(3.09)	47.3%	
35–44	228	1.65	(1.76)	24.1%		230	1.73	(1.79)	25.2%		230	5.00	(3.19)	42.6%	
45–54	193	1.36	(1.69)	18.1%		194	1.41	(1.66)	19.6%		191	4.38	(3.11)	35.6%	
55–64	250	1.26	(1.60)	16.8%		251	1.10	(1.54)	11.6%		253	4.17	(2.86)	31.2%	
65+	67	0.92	(1.24)	11.3%		68	1.06	(1.54)	11.1%		68	3.55	(2.93)	21.1%	
Prefer not to answer	34	0.97	(1.33)	16.7%		34	1.37	(1.92)	26.7%		33	4.13	(3.25)	36.7%	
**Gender**															
Male	907	1.48	(1.68)	21.2%	0.31	912	1.47	(1.72)	21.6%	0.19	908	4.10	(3.10)	37.2%	0.45
Female	104	1.67	(1.78)	20.2%		105	2.17	(1.82)	33.3%		105	5.38	(3.05)	45.7%	
Prefer not to answer	47	1.06	(1.31)	17.0%		47	1.47	(1.93)	23.4%		46	4.59	(3.12)	37.0%	
**Race/Ethnicity**															
American Indian or Alaska Native	18	0.78	(1.22)	11.1%	0.86	18	1.11	(1.91)	11.1%	0.76	17	2.47	(2.37)	11.8%	0.03
Asian	21	1.76	(2.19)	33.3%		21	1.19	(2.02)	19.0%		21	4.33	(3.50)	38.1%	
Black or African	23	1.43	(1.93)	17.4%		24	1.38	(1.66)	25.0%		24	4.42	(3.49)	33.3%	
Hispanic, Latinx, Spanish origin	24	1.88	(1.83)	29.2%		25	1.76	(2.13)	28.0%		25	4.68	(2.67)	32.0%	
White	805	1.52	(1.67)	20.5%		809	1.60	(1.74)	23.7%		807	4.75	(3.09)	38.2%	
Other or multiple race/ethnicity	82	1.16	(1.54)	18.3%		82	1.43	(1.85)	20.7%		81	4.53	(3.00)	39.5%	
Prefer not to answer	93	1.43	(1.64)	24.7%		93	1.28	(1.65)	19.4%		91	4.89	(3.21)	42.9%	
**Maritime Industry (vessel type)**															
Oceangoing cargo vessels	269	1.44	(1.66)	20.1%	0.02	268	1.46	(1.71)	20.5%	0.15	259	4.37	(3.04)	35.1%	0.02
Inland & coastal cargo vessels/workboats	421	1.28	(1.57)	16.9%		427	1.47	(1.68)	22.0%		418	4.66	(3.16)	37.3%	
Passenger vessels	78	1.31	(1.49)	16.7%		79	1.24	(1.63)	15.2%		79	4.19	(2.98)	26.6%	
Government/public vessels	330	1.78	(1.73)	27.0%		330	1.71	(1.80)	26.7%		325	5.01	(3.02)	43.4%	
Other vessels	86	1.37	(1.73)	16.3%		86	1.50	(1.89)	20.9%		84	4.90	(3.00)	39.3%	
**Credentials (Position)**															
Cadet/Pilot	56	1.20	(1.49)	16.1%	0.93	58	1.57	(1.63)	27.6%	0.36	56	4.77	(3.25)	44.6%	0.29
Licensed deck/engineer officer	817	1.49	(1.64)	20.4%		817	1.55	(1.72)	22.0%		803	4.74	(3.03)	38.0%	
Unlicensed deck/engineer	193	1.42	(1.72)	21.2%		195	1.41	(1.78)	23.1%		192	4.43	(3.28)	37.0%	
Other credentials	116	1.41	(1.66)	19.8%		118	1.46	(1.79)	21.2%		112	4.55	(2.92)	34.8%	
**All Respondents**	1184	1.46	(1.65)	20.4%		1190	1.52	(1.73)	22.4%		1165	4.68	(3.08)	37.9%	

* *p*-value obtained from one-way analysis of variance (ANOVA) on mean outcome scores.

**Table 3:** Summary of predictors included in regression models.

	*n*	%	Mean (SD)
COVID-19 concerns—total average *	**1123**		**2.92 (0.84)**
*Frequency of concern regarding:*			
Being away from family	1148		3.42 (1.22)
Mariner contract length	1145		2.82 (1.40)
Money	1142		2.93 (1.26)
Lack of work	1141		2.59 (1.31)
A family member getting COVID while I am away	1146		3.26 (1.27)
Myself getting COVID while at sea or ashore	1146		2.83 (1.28)
Extra work onboard if there is a COVID case on my vessel	1147		2.60 (1.30)
Vessel experiences—total average *	**1098**		**2.84 (1.03)**
*Frequency of experiencing:*			
Having no or inadequate access to Internet on a vessel	1124		2.99 (1.38)
Having no or inadequate cell service while in port, harbors, inland	1123		2.71 (1.31)
Being restricted in my ability to join or rejoin my family	1120		2.97 (1.38)
Having feelings of isolation aboard	1118		2.83 (1.37)
Having to quarantine due to a potential exposure to COVID	1118		2.50 (1.30)
Being denied shore leave when in port due to COVID	1122		3.33 (1.60)
Inability to join a vessel due to interruption of crew changes	1116		2.56 (1.40)
**Mariner impacts—total sum** *(check all that apply)*	**1234**		**2.51 (2.13)**
*Percent reporting:*			
Personnel have been infected with COVID	380	30.8%	
Port calls have been affected	583	47.2%	
Cargo onload/off load has been affected	297	24.1%	
Shore leave has been cancelled	684	55.4%	
Shore medical visits have been impacted	313	25.4%	
My mariner contract has been extended voluntarily	95	7.7%	
My mariner contract has been extended involuntarily	360	29.2%	
I have had restricted ability to get on or off the vessel	764	61.9%	
My vessel/contract has not been impacted due to COVID	202	16.4%	
**Longest time aboard a vessel without access to shore leave**	**1225**		
Less than 2 months	577	47.1%	
2 to 4 months	355	29.0%	
4 to 6 months	202	16.5%	
More than 6 months	91	7.4%	
Vessel support and safety culture—total average **	**1088**		**2.80 (0.66)**
*Level of agreement with statement:*			
If I am feeling stressed or sad, there is usually someone on the vessel I can talk to	1104		2.42 (0.89)
Vessel senior officers encourage the crew to get mental health help	1101		2.71 (0.89)
Senior officers encourage employees to work in accordance with safety and C19 rules	1100		3.26 (0.73)
Management support and safety culture **—total average	**1088**		**2.88 (0.78)**
*Level of agreement with statement:*			
Company management encourages the crew to get mental health help	1101		2.63 (0.93)
Management encourages employees to work in accordance with safety and C19 rules	1092		3.13 (0.81)
Job satisfaction—total average **	**1100**		**3.01 (0.54)**
*Level of agreement with statement:*			
I like the people I work with	1110		3.16 (0.63)
I like doing the things I do at work	1109		3.24 (0.61)
I get enough time to relax and recharge when on the vessel	1107		2.63 (0.84)
Self-reported physical health—total average ***	1165		3.38 (0.93)
Self-reported mental health—total average ***	1160		3.32 (1.10)
Self-reported sleep quality—total average ***	1157		2.72 (1.15)
**Days of physical activity/week**	1165		2.67 (1.07)
0 = No days	199	17.1%	
1 = 1–2 days	323	27.7%	
2 = 3–4 days	305	26.2%	
3 = 5 or more days	338	29.0%	

* Frequency scale: 1 = never, 2 = almost never, 3 = sometimes, 4 = fairly often, 5 = very often. ** agreement scale: 1 = strongly disagree, 2 = disagree, 3 = agree, 4 = strongly agree. *** Scale: 1 = poor, 2 = fair, 3 = good, 4 = very good, 5 = excellent; bold text indicates a summary measure

**Table 4:** Results of logistics and linear regression for outcomes of interest.

	PHQ-2 (*n* = 992)			GAD-2 (*n* = 996)			PSS-4 (*n* = 972)		
Predictors	OR	95% CI	*p*	OR	95% CI	*p*	Coeff.	95% CI	*p*
Age	1.06	0.92, 1.21	0.42	0.87	0.76, 0.99	0.04	−0.04	−0.15, 0.07	0.44
Credential (REF: cadet/pilot)									
Licensed deck or engineer officer	0.50	0.20, 1.38	0.16	0.35	0.15, 0.811	0.01	−0.51	−1.25, 0.24	0.19
Unlicensed deck or engineer	0.63	0.22, 1.89	0.40	0.45	0.18, 1.17	0.10	−0.33	−1.15, 0.49	0.43
Other credential	0.50	0.16, 1.68	0.26	0.40	0.14, 1.17	0.09	−0.63	−1.53, 0.26	0.17
Vessel (REF: oceangoing cargo vessel)									
Inland/coastal operations/workboats	1.39	0.80, 2.45	0.25	1.39	0.80, 2.41	0.24	0.67	0.23, 1.10	0.003
Passenger vessel operations	1.68	0.64, 4.17	0.28	1.09	0.42, 2.68	0.86	0.85	0.16, 1.54	0.02
Gov’t/public vessel operations	1.74	1.04, 2.95	0.04	1.49	0.89, 2.54	0.13	0.54	0.11, 0.97	0.01
Other type of vessel	1.26	0.52, 2.93	0.59	1.26	0.52, 2.89	0.60	0.93	0.27, 1.59	0.003
COVID-19 concerns	1.44	1.06, 1.95	0.02	1.93	1.43, 2.61	<0.001	0.54	0.30, 0.77	<0.001
Vessel experiences	1.62	1.22, 2.17	0.001	1.18	0.89, 1.56	0.24	0.30	0.06, 0.53	0.01
Mariner contract impacts	1.06	0.94, 1.20	0.32	1.05	0.93, 1.18	0.40	−0.02	−0.11, 0.07	0.73
Time without shore leave	0.98	0.77, 1.24	0.84	1.27	0.99, 1.63	0.06	0.05	−0.15, 0.25	0.64
Vessel support and safety culture	0.81	0.54, 1.24	0.33	0.69	0.46, 1.05	0.08	−0.45	−0.79, −0.10	0.01
Management support and safety culture	0.99	0.72, 1.37	0.95	0.99	0.72, 1.35	0.94	−0.09	−0.36, 0.17	0.49
Job satisfaction	1.06	0.68, 1.65	0.78	0.93	0.60, 1.44	0.76	−0.06	−0.41, 0.28	0.72
Self-reported physical health	0.83	0.64, 1.08	0.16	1.20	0.92, 1.56	0.17	0.01	0.19, 0.22	0.90
Self-reported mental health	0.38	0.29, 0.49	<0.001	0.27	0.21, 0.36	<0.001	−1.25	−1.44, −1.05	<0.001
Self-reported sleep quality	0.63	0.49, 0.81	0.0003	0.85	0.66, 1.08	0.18	0.02	−0.16, 0.20	0.79
Weekly physical activity	0.93	0.76, 1.13	0.41	0.88	0.93, 1.39	0.21	−0.13	−0.28, 0.03	0.10
Model R2			0.32			0.34			0.40

PHQ-2 and GAD-2 are logistic regression models, with the cut-off score of ≥3 used to indicate depression and anxiety, respectively, are likely; PSS-4 is linear regression model.

**Table 5:** Results of logistic and linear regression looking at frequency of COVID-19 concerns and outcomes of interest.

	PHQ-2 (*n* = 1037)			GAD-2 (*n* = 1042)			PSS-4 (*n* = 1022)		
Predictors *	OR	95% CI	*p*	OR	95% CI	*p*	Coeff.	95% CI	*p*
Being away from family	1.30	1.07, 1.58	0.007	1.40	1.16, 1.69	0.0004	0.38	0.19, 0.56	<0.0001
Mariner contract length	1.37	1.18, 1.59	<0.0001	1.10	0.96, 1.27	0.16	0.16	0.007, 0.32	0.04
Money	1.33	1.13, 1.57	0.0007	1.27	1.08, 1.49	0.004	0.31	0.14, 0.49	0.0005
Lack of work	1.10	0.94, 1.27	0.23	1.21	1.05, 1.40	0.01	0.28	0.12, 0.45	0.0008
A family member getting C19 while I am away	1.06	0.89, 1.26	0.52	1.12	0.95, 1.33	0.18	0.07	−0.11, 0.25	0.46
Myself getting C19 while at sea or ashore	0.99	0.84, 1.17	0.88	1.07	0.91, 1.25	0.42	0.09	−0.08, 0.27	0.31
Extra work onboard if there is a C19 case on vessel	1.10	0.95, 1.27	0.19	1.07	0.94, 1.25	0.25	0.19	0.03, 0.35	0.02

* All models include age, vessel type, and credential.

## Data Availability

The data underlying these analyses is available at https://github.com/bakermarissa/marinermentalhealth, accessed on 6 November 2022.
